# Study protocol: cluster randomised controlled trial to assess the clinical and cost effectiveness of a staff training intervention in inpatient mental health rehabilitation units in increasing service users’ engagement in activities

**DOI:** 10.1186/1471-244X-13-216

**Published:** 2013-08-28

**Authors:** Helen Killaspy, Sarah Cook, Tim Mundy, Thomas Craig, Frank Holloway, Gerard Leavey, Louise Marston, Paul McCrone, Leonardo Koeser, Maurice Arbuthnott, Rumana Z Omar, Michael King

**Affiliations:** 1 Mental Health Sciences Unit, University College London, Charles Bell House, 67-73 Riding House Street, London, W1W 7EJ, UK; 2 Centre for Health and Social Care Research, Faculty of Health and Wellbeing, Sheffield Hallam University, Montgomery House, 32 Collegiate Crescent, Collegiate Campus, Sheffield, S10 2BP, UK; 3 Centre for Professional and Organisational Development, Faculty of Health and Wellbeing, Sheffield Hallam University City Campus, Howard Street, Sheffield, S1 1WB, UK; 4 Health Service and Population Research Department, Institute of Psychiatry, King’s College London, London, SE5 8AZ, UK; 5 South London and Maudsley NHS Foundation Trust, Maudsley Hospital, Denmark Hill, London, SE5 8AZ, UK; 6 Bamford Centre for Mental Health and Wellbeing, University of Ulster, Northland Road, Derry, BT 48 7JL, UK; 7 Department of Primary Care and Population Sciences, University College, Rowland Hill Street, London, NW3 2PF, UK; 8 UCL PRIMENT Clinical Trials Unit, Research Department of Primary Care & Population Health, UCL Medical School, Royal Free Campus, Rowland Hill Street, London, NW3 2PF, UK; 9 Centre for the Economics of Mental and Physical Health, Institute of Psychiatry, King’s College London, De Crespigny Park, London, SE5 8AR, UK; 10 North London Service User Research Forum, Mental Health Sciences Unit, University College London, Charles Bell House, 67-73 Riding House Street, London, W1W 7EJ, UK; 11 Department of Statistical Science, University College London, Gower Street, London, WC1E 6BT, UK

## Abstract

**Background:**

This study focuses on people with complex and severe mental health problems who require inpatient rehabilitation. The majority have a diagnosis of schizophrenia whose recovery has been delayed due to non-response to first-line treatments, cognitive impairment, negative symptoms and co-existing problems such as substance misuse. These problems contribute to major impairments in social and everyday functioning necessitating lengthy admissions and high support needs on discharge to the community. Engagement in structured activities reduces negative symptoms of psychosis and may lead to improvement in function, but no trials have been conducted to test the efficacy of interventions that aim to achieve this.

**Methods/design:**

This study aims to investigate the clinical and cost-effectiveness of a staff training intervention to increase service users’ engagement in activities. This is a single-blind, two-arm cluster randomised controlled trial involving 40 inpatient mental health rehabilitation units across England. Units are randomised on an equal basis to receive either standard care or a “hands-on”, manualised staff training programme comprising three distinct phases (predisposing, enabling and reinforcing) delivered by a small team of psychiatrists, occupational therapists, service users and activity workers. The primary outcome is service user engagement in activities 12 months after randomisation, assessed using a standardised measure. Secondary outcomes include social functioning and costs and cost-effectiveness of care.

**Discussion:**

The study will provide much needed evidence for a practical staff training intervention that has potential to improve service user functioning, reducing the need for hospital treatment and supporting successful community discharge. The trial is registered with Current Controlled Trials (Ref ISRCTN25898179).

## Background

This research focuses on people with longer term mental health problems whose needs are such that they require inpatient rehabilitation. This group has complex, severe mental health problems that prevent them being discharged home following an acute admission. The majority have a diagnosis of schizophrenia [1] complicated by a range of additional problems such as treatment resistant (non-response to first line medications) which occurs in up to 30% [2], cognitive impairment (usually affecting executive function and verbal memory) and pervasive negative symptoms such as apathy, amotivation and blunted affect [3-5]. Co-existing problems such as substance misuse, premorbid learning disability and developmental disorders, such as those on the autism spectrum also affect some in addition to the primary psychosis [1], [6]. These kinds of complex problems contribute to major impairments in social and everyday functioning and challenging behaviours that impede recovery and increase the risk of adverse outcomes [6].

The proportion of people who experience complex mental health problems is relatively small. Around 10% of people newly referred to secondary mental health services require referral for rehabilitation [7] and, at any time, only 1% of mental health inpatients occupy a rehabilitation bed. In other words, this is a small volume, high needs group. However, as well as the significant clinical challenges they pose for professionals, their care constitutes a major resource pressure for the NHS and social services. Depending on what is included in the estimate, the costs associated with this group amount to 25-50% of the total mental health budget [8]. Identification of interventions that can reduce the need for inpatient care, even by a small reduction in length of stay, will have a large impact on the resources absorbed by this group (a reduction of one week in the mean length of stay on an inpatient rehabilitation ward represents a cost efficiency of around 2% of the annual budget).

Although there is good evidence for specific interventions (such as antipsychotic medication, cognitive behaviour therapy and family psychoeducation) that can improve outcomes for people with a diagnosis of schizophrenia [9], most people are referred for rehabilitation when the first-line treatment options have either been exhausted or where there are problems in engaging the person in treatments [6]. Despite the high levels of need of rehabilitation service users and the high costs of care for this complex group, there is currently very little evidence for effective interventions available to guide mental health rehabilitation practitioners. Understanding which approaches are best able to promote progress towards greater independence and successful community discharge is of obvious relevance clinically and in terms of better targeting resources to provide cost-effective services.

Due to the severe functional impairments of people with complex mental health needs, a common focus in rehabilitation services is occupational therapy with the aim of improving everyday living skills [10]. Although it has also long been known that facilitating service users’ activity reduces the negative symptoms of psychosis [11], [12], there is less clear evidence of its ability to improve social function, though some studies suggest an association through promoting motivation and daytime structure [13-15]. What is known is that the level of activity of users of acute inpatient services is alarmingly low: less than 17 minutes per day were spent in an activity other than sleeping, eating or watching TV in one survey in a London Trust [16], though there is very limited published data on the amount and types of activities undertaken in inpatient rehabilitation services. Shimitras et. al. [17] found that although users of these services spent more time sleeping than community rehabilitation service users, they also spent more time engaged in active leisure activities. Other studies have also found that people with schizophrenia spend a large amount of time engaged in passive activities such as sleeping and watching TV [18-20]. The last UK Government’s Social Exclusion Unit highlighted the role of education, training, volunteering, arts, leisure and sports in promoting community participation for mental health service users [21]. Although the importance of staff facilitation of service user activities has been highlighted [22], there have been no randomised controlled trials to test the efficacy of interventions that aim to achieve this. This study comprises one part of a five year national programme of research into mental health rehabilitation services in England, the Rehabilitation Effectiveness for Activities for Life (REAL) study.

### Research objectives

The aim of this study is to investigate the clinical and cost effectiveness of a staff training intervention (the “GetREAL” intervention) to increase service users’ engagement in activities.

The objectives are to:

•Investigate whether the GetREAL staff training intervention is associated with greater service user activity.

•Determine whether the GetREAL staff training intervention is associated with improved clinical outcomes.

•Examine whether the GetREAL staff training intervention is associated with improved social outcomes.

•Investigate whether the GetREAL staff training intervention is associated with improvement in the quality of mental health rehabilitation units.

•Investigate whether the GetREAL staff training intervention is a cost-effective approach to improving service users’ engagement in activities.

Our primary objective is based on ratings of service users’ activity 12 months after the staff training intervention is delivered, in order to understand whether it impacts on sustained change in the unit’s practices related to service user activities.

## Methods

### Trial design

This is a two-arm, cluster randomised trial in which inpatient mental health rehabilitation units are the unit of randomisation. The cluster design prevents potential “contamination” of the intervention between trial arms (if, for example, staff who had received the training at a unit randomised to receive the training were to move to a comparison unit). The trial has been approved by the South East Essex Research Ethics Committee (Ref. 09/H1102/45) and is registered with Current Controlled Trials (Ref ISRCTN25898179) http://www.controlled-trials.com/ISRCTN25898179.

### Study setting and sample

A survey of NHS inpatient mental health rehabilitation services across England was carried out during the first phase of the REAL research programme between 2009 and 2010 [23]. Most (52/60) NHS Trusts participated, comprising a total of 133 mental health inpatient rehabilitation units. These units were assessed using the Quality Indicator for Rehabilitative Care (QuIRC), an international, standardised quality assessment tool completed by the unit manager [24], [25]. Units scoring below the median on the total QuIRC score were eligible for inclusion in the trial. The median number of beds in these units was 12. Small units (those with fewer than eight beds) were excluded to i) ensure recruitment of adequate numbers of service users for whom outcome data would be available and ii) because we were concerned that the addition of two extra staff delivering the staff training intervention in small teams would represent a disproportionate increase in staffing compared to larger teams which would impact on the effect of the intervention (i.e. introduce a selection bias) and reduce the generalisability of the results. All service users within participating units were approached for potential participation. Data on those unable to give informed consent due to impaired mental capacity were collected from a key staff member and case notes.

## Study procedures

### Recruitment

Of the 133 services surveyed in 2009–2010, 64 score below the median on the QuIRC and were eligible for the trial. Units were randomly selected for potential participation by the study statistician (LM). Managers of these units were approached to explain the purpose of the trial. A study information sheet was sent to unit managers and they were given up to four weeks to decide if they wished their unit to participate. This gave adequate time for them to discuss any queries about the study procedures with the research team and to discuss the implications of participation with their own team. Units that agreed to participate were randomly allocated on an equal basis to receive the staff training intervention or to continue with usual care. Randomisation was staggered to allow sufficient time for the researchers to gather baseline data and for the delivery of the staff training intervention sequentially at each unit. Randomisation was carried out independently of the research team by the Aberdeen Randomisation Service.

### Study interventions

Intervention units: Units allocated to this arm receive the GetREAL staff training intervention. The intervention was developed initially by SC, CH, TM, HK, FH, TC and MA and further developed through a consultation event with mental health occupational therapists from across England, and through piloting in two units. The intervention comprises three phases (predisposing; enabling; reinforcing). The Predisposing stage aims to facilitate a focus on the need for change and gain local service “sign up” [26]. The Enabling stage involves identifying and removing barriers to change, team-level action planning and the development of new necessary skills [27]. The Reinforcing stage involves maintaining changes once they are in place, identifying and implementing team changes and monitoring approaches in order to reinforce sustainable change [28]. The staff training intervention has been endorsed by the UK College of Occupational Therapy.

The predisposing stage comprises a consultation meeting with senior service managers and senior clinicians to explain the purpose of the staff training programme and gain support, facilitated by a member of the trial management group (HK, FH or TC). The enabling and reinforcing stages are delivered by one of two GetREAL intervention teams comprising a senior occupational therapist, an activity worker and a service user. The occupational therapist and activity worker spend five weeks in each unit. During the first week they review the unit’s resources and practices related to service user engagement. Along with the service user member, they facilitate a one day training course for all nurses and unqualified staff of the unit. The content is tailored to resources in each service and demonstrates motivational techniques [29], [30] and occupational therapy techniques to encourage service users’ engagement in activities.

The occupational therapist and activity worker work with staff in the unit daily for the rest of the five weeks to model and give intensive, hands on support for staff to gain confidence in the implementation of the techniques and interventions learned during the training course. The reinforcing stage starts during the fifth week, when the GetREAL team facilitates a half day workshop to review the intervention with the service manager and staff and agree how best the skills acquired can be incorporated into the unit’s usual structures and processes. An Action Plan reflecting this is drawn up by the GetREAL team’s occupational therapist and a member of staff is identified who will oversee delivery of the Action Plan in the unit after the GetREAL team have left. Email support to the unit is available from the GetREAL team over the next 12 months. If no contact is made by the unit, a prompt email is sent by the GetREAL team occupational therapist every six months during this period to encourage contact.

Comparison units: Units allocated to this arm continue with their usual service and are able to use any resources at their disposal to provide maximum care for service users. There are no restrictions on the work of these teams.

### Treatment fidelity

At the end of each unit’s intervention, the supervising occupational therapist completes a proforma together with the staff training teams’ occupational therapists and a senior member of the research team who attended the predisposing meetings. This proforma records the delivery of 24 specific aspects of the GetREAL intervention. Each item completed achieves a score of 1, giving a total possible score of 24 (see Table 1).

**Table 1:** GetREAL staff training intervention fidelity assessment

**Get REAL intervention component**	**Completed (Y/N)**
**Predisposing Visit**	
Predisposing meeting held with the unit’s senior team members attended by at least one of the REAL research steering group’s senior psychiatrists (HK, FH, TC) to explain the purpose of the GetREAL intervention and gain senior staff “sign up” to support the GetREAL team’s work	
Dates for the first GetREAL training day/s for unit staff, and release of staff to attend, are agreed with the unit manager before the GetREAL team arrive	
Unit manager agrees to provide unit keys and, where possible, IT access/email accounts for the GetREAL team OT and Activity Worker	
**Initial Training**	
At least two members of the GetREAL team deliver the initial training	
At least 50% of the unit staff attend	
Initial evaluation forms are completed by all staff attending	
Action plans are agreed for the next 4 weeks	
**Enabling Phase**	
GetREAL team work alongside unit staff for at least 5 weeks including the training days	
At least one structural change/enhancement is agreed to facilitate service users’ (SU) activities	
Note whether any other unit structural/process changes made secondary to the GetREAL team’s suggestions that may not directly relate to SU activities	
Individual SU goal setting (regarding activities) is carried out and recorded in care plans for at least 50% of SUs on the unit	
**Final Training**	
At least two members of the GetREAL team deliver the final training	
At least 50% of the unit staff attend	
The certificate of attendance is awarded to at least 50% of unit staff (staff have to attend both the initial and final training to receive the certificate)	
**Sustainability and Reinforcing Phase**	
At the end of the 5 weeks, a written action plan for the unit to continue the GetREAL work for the next 12 months is agreed	
The 12 month action plan is circulated to all unit staff by the GetREAL team	
At the end of the 5 weeks, activity is included in at least 50% of SUs’ individual care plans	
A link person is identified to keep email contact with the GetREAL team/steering group members for up to 12 months	
GetREAL team/steering group members make email contact at least twice with the unit in the 12 months following the 5 week visit	
The link person contacts the GetREAL team at least once during the 12 month period	
**Supervision and Support of the GetREAL Team**	
GetREAL SU consultants are supported by the OTs through face to face/email/telephone discussion as required	
GetREAL Activity Workers are supervised by the OTs weekly during each intervention period	
GetREAL OTs are supervised at least three times per intervention period by the REAL research OT and/or the REAL organisational change psychologist by phone, skype, email or face to face contact	
GetREAL OTs attend line management meeting with the REAL senior OT once per intervention period	
**Total Score = total number of Y’s (max 24)**	

## Measures

Outcomes are assessed 12 months after baseline data collection. Baseline data are collected in four units (two allocated to receive the GetREAL intervention and two comparison units) by the researchers within the four week period prior to the GetREAL teams starting their intervention. All service users within each unit are eligible to participate in the study. The researchers approached all service users to explain the purpose and process of the study and a participant information sheet was given to them. They were given the opportunity to ask any questions about the study. Those that were assessed having capacity to give informed consent and declined to participate were not interviewed. This process is repeated for baseline and follow-up data collection.

### Primary outcome

The primary outcome is the degree to which service users are engaged in activity over a given week as assessed using the Time Use Diary [31]. This measure assesses service users’ activities over the previous week during four periods each day; morning, lunchtime, afternoon and evening. The degree of engagement in activity as well as the complexity of the activity is rated on a scale of 0 to 4 for each time period, giving a maximum possible score of 112. The diary is completed retrospectively during a structured interview with the service user. The scale has demonstrated good inter-rater reliability and has been validated [31]. If service users lack capacity to give informed consent to participate in a face to face interview, information about their activities in the preceding week is gathered from the case records and discussions with their primary nurse.

### Secondary outcomes

i) Service users’ social functioning as rated by a key staff member using the Life Skills Profile [32]. This measure comprises 39 staff rated items each rated on a four point likert scale with the most positive response scoring 4 and the least scoring 1, giving an overall score ranging between 39 and 156.

ii) length of admission

iii) percentage of service users discharged/ready for discharge in the last 12 months

iv) percentage of service users discharged to an out of area placement in the last 12 months

v) staff attitudes towards service user’s progress are assessed using the question “I expect this person to be able to move on to a more independent setting within the next 12 months”. The response is in the form of a five point likert scale.

vi) service quality as assessed using the Quality Indicator for Rehabilitative Care (QuIRC) [24], [25]. This tool comprises 145 questions on service quality and provision (e.g. number of beds, average length of stay, treatments and interventions, staffing, staff turnover, training and supervision, links with community resources such as colleges, employment agencies and leisure facilities, service user involvement in care planning and running the unit, promotion of service users’ independent living skills, the protection of service users’ human rights such as privacy and dignity, legal rights and the use of restraint and seclusion. The QuIRC gives percentage ratings on seven domains of care: Living Environment, Therapeutic Environment, Treatments and Interventions, Self-management and Autonomy, Social Interface, Human Rights and Recovery Based Practice.

### Baseline data

Descriptive data on all service users are collected from staff and case notes as follows: demographics (age, gender, ethnic group); diagnosis; length of history; length of current admission. Primary and secondary outcomes measures are completed as described above. Potential mediators of outcomes are assessed including the staffing of the unit, recorded from the unit manager, service users’ substance use, assessed using the staff rated Clinician Alcohol and Drug Use Scales [33] and challenging behaviours which may make community placement difficult, assessed using the staff rated Special Problems Rating Scale [34].

### Costs of care

Data on costs of care are collected using an adapted form of the Client Services Receipt Inventory [35] which measures service users’ contacts with staff, and data on the unit’s budget collected from the unit manager. Cost data are collected at baseline and 12 month follow-up.

### Qualitative component

We added a qualitative component to the study in order to obtain: (a) an understanding of rehabilitation staff receptivity to the intervention and those delivering it; (b) elements considered to be most beneficial or unhelpful; and (c) barriers and facilitators in maintaining the intervention over time. These areas will be explored through individual interviews with 2–3 service users, and focus groups with staff, in units that receive the GetREAL intervention, purposively selected on the basis of size and regional location across England. Topic guides for staff focus groups and service user interviews will be used to ensure the same areas are covered, with new topics or issues that emerge during the process being added for subsequent groups/interviews.

## Data management

Data are entered into an Access database by the researchers. Range and logic checks have been built in to assist with data cleaning. Ten percent of data will be double entered to check for data entry errors with an error rate set at 5%, above which all data would be double entered.

Qualitative data are digitally recorded and transcribed. Transcripts are imported to specialist software (Atlas Ti 6) for analysis.

## Power and sample size

Our primary analysis is based on a comparison of two means (the mean unit score on the Time Use Diary at 12 month follow-up). To detect an effect size of 0.35 SD between the intervention and comparison groups, with 80% power and assuming an ICC of 0.04 and an average cluster size of 12, we require 186 patients in each arm from a minimum of 31 clusters (rehabilitation units).

## Data analysis

We shall follow CONSORT guidelines for the analysis of randomised trials and for the presentation of our results.

### Statistical analysis

The baseline characteristics of the service users will be summarised using mean (SD), median (interquartile ranges) or proportions as appropriate and compared across the trial arms descriptively. Random effects linear regression will be used for the primary outcome adjusted for the baseline value of the Time Use Diary score to evaluate the effect of the intervention. Some of the service users will be different to those present at baseline as some present at baseline will have been discharged and new service users will have been admitted. Therefore, the mean baseline score calculated for each unit (based on the service users present in the unit at the baseline data collection time point) will be used in the model rather than the scores for the individual service users. Bias due to missing data and predictors of missingess will be investigated. The analysis will be adjusted for the predictors of missingness that are associated with the outcome if required, to preserve the missing at random mechanism in the data. Assumptions of normality of the residuals will be investigated. For service users with missing primary outcome data (due to lack of capacity to give informed consent to participate in the interview), the agreement between the staff and service user Time Use Diary scores will be examined by plotting the two scores against each other. If the data roughly form a straight line, then the staff diaries will be substituted for the service users’ diaries as part of a sensitivity analysis. Otherwise the missing Time Use Diary scores will be imputed using multiple or regression imputation. A sensitivity analysis will also be carried out adjusting for the length of admission in the unit at the 12 month follow-up and the GetREAL intervention fidelity score.

For the secondary outcomes, appropriate statistical models allowing for clustering will be used for outcomes measured at the service user level and appropriate statistical tests based on the cluster summary measures will be used for outcomes measured at the unit level. The results from the secondary analyses will be treated as exploratory and only estimates and confidence intervals will be reported. All analyses will be carried out on an intention to treat basis.

A full statistical analysis plan will be developed by the study statisticians in collaboration with the research team and ratified by in Independent Trial Steering Group nearer the analysis stage.

### Cost effectiveness of the GetREAL intervention

Costs of care at follow-up are calculated by combining service use data collected using the Client Service Receipt Inventory combined with national unit costs and an estimate of the extra resources required for the GetREAL staff training. Cost-effectiveness of the GetREAL intervention will be assessed by combining service costs with the primary outcome (assessed using the Time Use Diary [31]) using an incremental cost-effectiveness ratio. This will show the extra cost incurred for the intervention to achieve an extra 1% of time spent in activities.

There will be uncertainty in the cost-effectiveness results obtained which we will address using cost-effectiveness planes. This will involve producing a large number of estimates of cost and outcome differences using bootstrapped regression models and plotting each pair of differences in the form of a scatter plot. This will indicate the likelihood that compared to the comparison group the intervention produces (i) higher costs and worse outcomes, (ii) higher costs and better outcomes, (iii) lower costs and worse outcomes, and (iv) lower costs and better outcomes. In addition, we will conduct sensitivity analyses around key unit costs to see what impact changing these has on the total costs and cost-effectiveness.

### Qualitative data analysis

We will adopt a straightforward approach to the qualitative data analysis, using a standard thematic procedure which will be overseen by GL and HK. The transcripts will be read and coded by the researchers using the topic guide as the initial coding frame. Further codes will be added as additional themes emerge. Validity and reliability of coding will be checked by GL and HK who will recode a random sample of 2–3 focus group and service user interview transcripts. We will use conceptual maps to explore relationships and connections in the data and develop specific questions to investigate in further analyses.

## Methods to protect against bias

### Response

Our primary outcome data is, ideally, collected through a face to face interview that the researcher carries out with service users. This will minimise any bias that would ensue if we were to ask nursing staff to complete the activity diaries. However, since some service users may not be able to give informed consent to participate in a face to face interview due to the severity of their symptoms, we gained approval from the SE Essex Research Ethics Committee to gather data from case notes and staff on service users who lacked capacity to give informed consent for participation. We are therefore able to gather information about their activities over the preceding week from the case records and discussions with a key staff member in the clinical team.

All secondary outcome measures are researcher or staff rated. Complete data collection on all consenting participants and those unable to give informed consent will therefore be possible for all primary and secondary outcome analyses. Only service users who have capacity to decline consent (and do so) and service users on leave from the unit and unavailable for interview are unable to be included.

### Unmasking of researchers

We have stressed that the unit staff should not reveal to the researchers whether they received the GetREAL training intervention. Any unmasking of researchers is reported to the programme management group. We will assess the degree of unmasking by asking the researchers to record their view about which units received the intervention and which were comparison sites after they collect follow-up data.

### Loss to follow-up

Since we are assessing all service users present in each unit at 12 months after randomisation, loss to follow-up of service users assessed at baseline is not an issue. However, the economic downturn is having an impact on NHS resources leading to some mental health rehabilitation units being considered for closure. Although this has not affected any of the units participating to date, we have increased the number of units recruited from 35 to 40 to ensure we have enough units in the study at 12 month follow-up to allow for data to be gathered on at least 372 service users (186 per trial arm).

## Discussion

This is the first large scale randomised controlled trial to investigate the clinical and cost-effectiveness of a staff training intervention in mental health rehabilitation units aimed at improving service users’ engagement in activities. Given the paucity of evidence based guidance available to help clinicians in treatment of this complex service user group, the results are likely to be of national and international interest. They will feed into the limited evidence base related to mental health occupational therapy and potentially guide investment in these services. Although we acknowledge that it is challenging to assess outcomes 12 months after the staff training intervention, we feel this is appropriate since any changes in practice facilitated by the intervention that impact positively on service users’ engagement in activities need to be sustainable beyond the training intervention to justify a national rollout of the intervention.

## Competing interests

The REAL study has been funded by the Programme Grants for Applied Research funding scheme of the National Institute of Health Research England (ref RP-PG-0707-10093). The costs of publication of this manuscript will be covered from this grant. The National Institute of Health Research will not gain or lose financially from the publication of this manuscript, either now or in the future. The views expressed are those of the authors and not necessarily those of the National Health Service, the National Institute for Health Research or the Department of Health in England.

## Authors’ contributions

HK, MK, FH, TC, SC, TM and MA conceived and designed the study. RO, LM, MK and HK designed the quantitative data analysis strategy, PMcC and LK designed the health economic components of the study and GL developed the qualitative components. All authors were involved in drafting and reviewing the manuscript and agreeing its final content before submission.

## Pre-publication history

The pre-publication history for this paper can be accessed here:

http://www.biomedcentral.com/1471-244X/13/216/prepub
